# Effects of Kongsheng Zhenzhong Pills on Neuroinflammation and cGAS-STING Pathway Activity in BV-2 Microglia

**DOI:** 10.3390/biomedicines14081665

**Published:** 2026-07-24

**Authors:** Han Zhang, Dongfeng Wei, Xuejie Han, Wei Wu, Xingfang Liu, Manman Xu

**Affiliations:** 1 Geriatric Department, Guang’anmen Hospital, China Academy of Chinese Medical Sciences, Beijing 100053, China; zhanghan6760@163.com (H.Z.); ww223841@163.com (W.W.); 2 Institute of Basic Research in Clinical Medicine, China Academy of Chinese Medical Sciences, Beijing 100700, China; weidongfeng@aliyun.com (D.W.); xuejieh@126.com (X.H.); 3 Research Department, Swiss University of Traditional Chinese Medicine, 5330 Bad Zurzach, Switzerland; xingfang.liu@tcmuni.ch

**Keywords:** Kongsheng Zhenzhong Pills (KSZZP), cGAS-STING pathway, neuroinflammation, microglia, oxidative stress

## Abstract

**Background**: Global aging has led to a rise in neurodegenerative diseases. Neuroinflammation constitutes a core pathological mechanism in these disorders. The Kongsheng Zhenzhong Pill (KSZZP), a modern preparation derived from a classical Chinese herbal formula, are reputed for their sedative and cognition-enhancing effects. However, the molecular mechanism underlying its modulation of microglia-mediated neuroinflammation remains unclear. The present study was designed to assess the intervention effects of KSZZP on LPS-induced neuroinflammation in BV-2 microglial cells and to preliminarily explore the potential molecular mechanisms involved. **Methods**: An in vitro neuroinflammation model was established in LPS-induced BV-2 microglial cells. The chemical components of KSZZP were identified using UPLC-Q-Exactive HFX technology. The pharmacological effects of KSZZP were evaluated by assessing cell activation, inflammatory response, oxidative stress, and apoptosis. Molecular docking and Western blotting were used to explore the specific mechanism of its action on the cGAS-STING pathway. **Results**: Chemical analysis identified 67 components in KSZZP, primarily flavonoids, prenyl lipids, and isoflavones. KSZZP treatment dose-dependently inhibited LPS-induced BV-2 microglial activation and significantly reduced pro-inflammatory factor release. Furthermore, it alleviated oxidative stress, mitigated mitochondrial ultrastructural damage, and inhibited apoptosis induced by LPS. Molecular docking revealed that key active components of KSZZP exhibit strong binding potential to cGAS and STING proteins. Western blotting further confirmed that KSZZP dose-dependently suppressed the expression of key cGAS-STING pathway proteins (cGAS, STING) and downstream proteins associated with M1 polarization (iNOS, TNF-α, COX-2). **Conclusions**: This study indicates that KSZZP alleviates LPS-induced microglial activation, neuroinflammation, oxidative stress, mitochondrial damage, and apoptosis, and these effects may involve the modulation of the cGAS-STING signaling pathway. Collectively, these findings provide a preliminary experimental basis for understanding the anti-neuroinflammatory mechanism of KSZZP and support its potential application in the prevention and treatment of neurodegenerative diseases.

## 1. Introduction

Global population aging is accelerating, leading to a rise in major public health challenges worldwide, such as Alzheimer’s disease (AD) and Parkinson’s disease (PD) [1]. The pathogenesis of these diseases is complex and encompasses multiple pathological processes, such as neuroinflammation, oxidative stress, mitochondrial dysfunction, and apoptosis [2]. Among these, neuroinflammation is recognized as a core mechanism. Within this context, microglia—the resident primary immune cells of the central nervous system—play a crucial role [3]. Under pathological conditions, microglia become overactivated and adopt a pro-inflammatory M1 phenotype. This activation leads to the release of large quantities of inflammatory factors, including tumor necrosis factor-α (TNF-α) and interleukin-1β (IL-1β), and reactive oxygen species (ROS), which directly mediate neuronal damage and synaptic dysfunction [4,5]. The levels of inducible nitric oxide synthase (iNOS)—a characteristic M1 microglial marker—and its product nitric oxide (NO) are key indicators for assessing neuroinflammation [6].

Currently, clinical treatment options for neurodegenerative diseases are limited. Available drugs primarily offer symptomatic relief and are frequently inadequate for halting disease progression. Direct interventions targeting neuroinflammation, such as long-term use of non-steroidal anti-inflammatory drugs, are associated with risks of gastrointestinal and cardiovascular side effects [7]. In this context, TCM, which features a multi-component, multi-target holistic regulatory approach, offers novel perspectives and unique potential for managing such complex diseases. Kongsheng Zhenzhong Pills (KSZZP) are derived from the renowned historical formula “Kongsheng Zhenzhong Dan,” documented in Sun Simiao’s Tang Dynasty treatise Beiji Qianjin Yaofang (Volume 14) [8]. The modern pill formulation consists of four herbs: Tortoise Shell, Dragon Bone, *Acorus tatarinowii*, and *Polygala tenuifolia*. KSZZP are recognized for their effects in tonifying the heart and kidneys, improving cognitive function, and calming the spirit. Clinically, they are commonly used to treat conditions associated with cognitive dysfunction [9]. Studies on the individual constituent herbs have confirmed their neuroprotective and anti-inflammatory potential, demonstrating an ability to ameliorate neurological damage [10,11,12]. However, the overall mechanism of action of KSZZP—particularly how it exerts therapeutic effects through the regulation of microglia-mediated neuroinflammation-and its specific molecular targets remain unclear and warrant in-depth investigation.

The LPS-stimulated BV-2 microglial cell model is among the most extensively utilized in vitro models for investigating neuroinflammatory mechanisms [13]. In addition to activating microglia through canonical pathways such as TLR4/NF-κB and the NLRP3 inflammasome, emerging evidence indicates that LPS can induce mitochondrial dysfunction, leading to the leakage of mitochondrial DNA (mtDNA) into the cytosol, which in turn activates the cGAS-STING pathway [14,15]. The cGAS-STING signaling pathway, a core component of innate immunity, has attracted growing interest in recent years for its role in neuroinflammation and neurodegenerative diseases [16,17]. This pathway constitutes a crucial cytosolic DNA-sensing mechanism. Upon activation by cytoplasmic double-stranded DNA (e.g., from neuronal injury or mitochondrial stress), it drives the expression of type I interferons and various inflammatory factors. Consequently, it plays a key role in initiating and amplifying neuroinflammation and can contribute to apoptosis [18]. Studies indicate that inhibiting the cGAS-STING pathway mitigates microglial polarization to the M1 phenotype and alleviates neuroinflammatory responses, thereby exerting neuroprotective effects [19]. Evidence clearly establishes the involvement of the cGAS-STING pathway in the pathological progression of neurodegenerative diseases [20,21]. Consequently, targeted inhibition of the cGAS-STING pathway to modulate neuroinflammation has emerged as a promising therapeutic strategy for neurodegenerative diseases.

This study aims to investigate the inhibitory effect of KSZZP on LPS-induced microglial overactivation and neuroinflammatory responses, and to provide a preliminary exploration of the possible molecular mechanisms. We employed an LPS-induced BV-2 microglial cell model and integrated techniques including cellular assays, molecular docking, and Western blotting. This work aims to elucidate the scientific basis of the formula’s traditional “cognition-enhancing and sedative efficacy” from a modern pharmacological perspective. The findings are expected to provide experimental evidence supporting the expanded application of KSZZP in preventing and treating neurodegenerative diseases.

## 2. Materials and Methods

### 2.1. Reagents and Chemicals

The BV-2 microglial cell line (FH0355) and its specific culture medium (FH-Bv-2), which consists of Dulbecco’s Modified Eagle Medium (DMEM) supplemented with 10% fetal bovine serum (FBS) and 1% penicillin/streptomycin (P/S), were purchased from Shanghai Fuheng Biotechnology Co., Ltd. (Shanghai, China). Fetal bovine serum (F-001-A) was provided by Suzhou Yanjie Biotechnology Co., Ltd. (Suzhou, China). Phosphate-buffered saline (PBS, G4202) and 0.25% trypsin (G4004) were obtained from Wuhan Servicebio Technology Co., Ltd. (Wuhan, China). RIPA lysis buffer (JC-PL001) and SDS-PAGE gel kits (TC-PE022/R) were purchased from Beijing Jingcai Biotechnology Co., Ltd. (Beijing, China). Phenylmethylsulfonyl fluoride (PMSF, P0100) was purchased from Beijing Solarbio Science & Technology Co., Ltd. (Beijing, China). Protein marker (PE061) was obtained from Beijing Zhonghui Hecai Biotechnology Co., Ltd. (Beijing, China). The Apoptosis Detection Kit (A211-01) was purchased from Vazyme Biotech Co., Ltd. (Nanjing, China). The reactive oxygen species assay kit (CB10366), nitric oxide assay kit (CB10523), Cell Counting Kit-8 (CCK-8, C0037), malondialdehyde (MDA) assay kit (CB10205), superoxide dismutase (SOD) assay kit (CB10200), glutathione (GSH) assay kit (CB10323), BCA protein assay kit (P0012), Mouse IL-6 Uncoated ELISA kit (CB10187), Mouse IL-1β Uncoated ELISA kit (CB10173), and Mouse TNF-α Uncoated ELISA kit (CB10851) were all purchased from Shanghai Keaibo Biotechnology Co., Ltd. (Shanghai, China). Antibodies against Iba1 (DF6442), iNOS (AF0199), TNF-α (AF7014), GAPDH (AB2302), and COX-2 (AF7003) were purchased from Affinity Biosciences (Cincinnati, OH, USA). Antibodies against cGAS (26416-1-AP) and STING (19851-1-AP) were purchased from Wuhan Sanying Biotechnology Co., Ltd. (Wuhan, China). All reagents used were chromatography grade products supplied by ANPEL Laboratory Technologies (Shanghai) Inc. (Shanghai, China), specifically including methanol (CAEQ-4-003302-1000), acetonitrile (CAEQ-4-000308-4000), isopropanol (CAEQ-4-000309-4000), and formic acid (CAEQ-4-000313-0050).

### 2.2. Experimental Methods

#### 2.2.1. Preparation of Kszzp Solution

Kongsheng Zhenzhong Pills (KSZZP) were obtained from Tongrentang Pharmaceutical Factory, Beijing Tongrentang Co., Ltd. (Beijing, China) (National Drug Approval No. Z20026412). All experiments in this study were performed with a single production batch of KSZZP to ensure compositional consistency and minimize inter-batch variability. A voucher specimen (No. KSZZP-2025-001) has been deposited at the Medical Experiment Center, China Academy of Chinese Medical Sciences, and is available on request. KSZZP was ground and sieved through an 80-mesh sieve to obtain fine powder. Then, 100 mg of the fine powder was accurately weighed and mixed with 0.2 mL of DMSO, 0.2 mL of anhydrous ethanol, and 0.6 mL of ultrapure water to yield a 1 mL mixed solution (vehicle composition: 20% DMSO, 20% ethanol, and 60% water, *v*/*v*/*v*). The mixture was sonicated at room temperature for 1 h to achieve complete dissolution and dispersion. After centrifugation at 4 °C and 5000 rpm for 10 min, the supernatant was collected, and the pellet was discarded. Finally, the supernatant was filter-sterilized through a 0.22 μm organic phase membrane, aliquoted, and stored as the KSZZP stock solution (approximately 100 mg/mL). It should be noted that the KSZZP concentrations reported in this study were calculated based on the total weight of the pulverized pill material and thus represent the concentration of the total pill extract rather than that of individual purified herbal compounds. This approach is based on the consideration that, as a commercially manufactured multi-herbal formulation, the therapeutic effects of KSZZP arise from the synergistic actions of multiple constituents. Therefore, using the total extract weight as the basis for determining concentrations in cellular assays allows a more accurate reflection of the overall pharmacological effects of the formulation.

#### 2.2.2. Component Identification

**Preparation of the test sample solution:** Precisely 50 mg of KSZZP fine powder was mixed with 1 mL of extraction solvent (water: acetonitrile: isopropanol = 1:1:1, *v*/*v*/*v*). The mixture was vortexed for 60 s and then subjected to low-temperature sonication for 30 min for extraction. After centrifugation at 4 °C (12,000 rpm, 10 min), the supernatant was collected, kept at −20 °C for 1 h, and then centrifuged again under the same conditions. The supernatant was then dried under vacuum. The resulting residue was reconstituted in 200 μL of 50% acetonitrile, vortexed, and centrifuged at 4 °C (14,000 rpm, 15 min). The final supernatant was collected and used as the test sample solution.

**UPLC-Q-Exactive HFX analysis:** Analysis was performed using an ultra-high-performance liquid chromatography (UPLC) system (Vanquish, Thermo Scientific, Waltham, MA, USA) coupled to a high-resolution Q Exactive HFX mass spectrometer (Thermo Scientific, USA). Chromatographic separation was achieved on a Waters HSS T3 column (100 mm × 2.1 mm, 1.8 μm) maintained at 40 °C. A mobile phase of 0.1% (*v*/*v*) aqueous formic acid (A) and 0.1% (*v*/*v*) formic acid in acetonitrile (B) was employed for gradient elution at a flow rate of 0.3 mL/min. The gradient program was as follows: 0–1 min, 0% B; 1–12 min, 0% → 95% B; 12–13 min, 95% B; 13.1 min, 95% → 0% B; 13.1–17 min, 0% B. Mass spectrometric detection utilized an electrospray ionization (ESI) source operating in positive-negative switching mode. The ion source and transfer tube temperatures were set to 350 °C and 320 °C, respectively. The spray voltages were +3.0 kV (positive ion mode) and −2.8 kV (negative ion mode). The sheath gas and auxiliary gas flow rates were set to 40 and 10 arbitrary units (arb), respectively. Full MS/dd-MS^2^ scans were acquired over an *m*/*z* range of 70–1050. The full MS and MS/MS resolutions were set to 70,000 and 17,500, respectively. All samples were maintained at 4 °C in the autosampler tray and injected in a randomized order. Quality control (QC) samples were interspersed throughout the analytical sequence to monitor system stability and data reliability.

**Data preprocessing:** Raw data were processed using Progenesis QI software (Version 3.1; Waters Corporation, Milford, MA, USA). This included baseline filtering, peak detection, peak alignment, retention time correction, and intensity normalization to generate a data matrix of retention time, *m*/*z*, and peak intensity. Components (features) with MS/MS spectra were identified by matching against a custom-built traditional Chinese medicine MS/MS spectral library and applying known fragmentation rules.

#### 2.2.3. Cell Culture

BV-2 cells were routinely cultured in DMEM supplemented with 10% FBS and 1% P/S (FH-Bv-2) and maintained at 37 °C in a humidified 5% CO_2_ incubator. The medium was replaced every other day. Upon reaching 80–90% confluence, cells were passaged using 0.25% trypsin. Cells in the logarithmic growth phase were harvested for subsequent experiments. All experiments were performed with BV-2 cells at passages 4–10 (P4–P10) post-thawing; cells were not used beyond passage 15. To ensure experimental consistency and reproducibility, fresh cells were thawed from liquid nitrogen stocks for each independent experiment. Cell morphology was regularly monitored under an inverted microscope throughout the experimental period to confirm that the cells retained the typical microglial morphology (amoeboid or bipolar) and exhibited no abnormal changes.

#### 2.2.4. Cell Viability and Ec_50_ Determination

Cell viability was assessed using the Cell Counting Kit-8 (CCK-8) assay. Cells were seeded in 96-well plates at a density of 4 × 10^3^ cells/well. Two experimental paradigms were conducted: first, to assess direct cytotoxicity, cells were treated with a range of KSZZP concentrations (19.53–2500 μg/mL) for 24 or 48 h under basal conditions; second, to evaluate the protective effect of KSZZP, an inflammatory model was established by stimulating cells with 1 μg/mL LPS [22,23,24]. Each treatment group consisted of five replicate wells. At 1.5 h prior to the end of the treatment period, 10 μL of CCK-8 solution was added to each well. After incubation for 1–4 h, absorbance was measured at 450 nm, and cell viability was calculated. The half-maximal effective concentration (EC_50_) of KSZZP was determined using non-linear regression analysis (e.g., with GraphPad Prism). Based on these results, 40 μg/mL was selected as the low dose and 160 μg/mL as the high dose for subsequent mechanistic studies.

#### 2.2.5. Assessment of Microglial Activation

##### Immunofluorescence Staining

To observe microglial morphology, cells were seeded on sterile coverslips placed in 24-well plates. Based on the dosing regimen from Section 2.2.4, cells were assigned to the following groups: Control group (equal volume of vehicle, i.e., ultrapure water containing 20% DMSO and 20% ethanol), Model group (1 μg/mL LPS + equal volume of vehicle), KSZZP-L group (1 μg/mL LPS + 40 μg/mL KSZZP), KSZZP-H group (1 μg/mL LPS +160 μg/mL KSZZP), and C-176 group (1 μg/mL LPS + 5 μM C-176). All groups were adjusted to the same final vehicle concentrations (0.032% DMSO and 0.032% ethanol, *v*/*v*) by supplementation with vehicle as needed. This concentration is far below that commonly used as a vehicle control in BV-2 microglial cells (0.1% DMSO) [25] and is also lower than concentrations reported to affect inflammatory responses in BV-2 cells (0.5–1% ethanol) [26]. Therefore, the vehicle control (Control group) served as an appropriate baseline for assessing the effects of LPS and KSZZP. Following treatment, cells were fixed with 4% paraformaldehyde, permeabilized with 0.1% Triton X-100, and blocked with 5% BSA. Cells were then incubated overnight at 4 °C with a rabbit anti-Iba-1 primary antibody (1:200). This was followed by incubation with a Cy3-conjugated goat anti-rabbit IgG secondary antibody (1:500) for 1 h at room temperature in the dark. Nuclei were counterstained with DAPI. After mounting, images were acquired using a laser scanning confocal microscope and analyzed for cell morphology, as well as the expression and localization of Iba-1.

##### Flow Cytometry

To quantify microglial activation, BV-2 cells were seeded in 6-well plates and treated as per the experimental groups. Cells were harvested by trypsinization, fixed with 4% paraformaldehyde, and permeabilized with 90% ice-cold methanol. Cells were then stained in the dark with fluorescently labeled antibodies: anti-CD11b-FITC and anti-Iba-1-PE. A minimum of 10,000 events per sample were acquired on a flow cytometer. Data were analyzed using FlowJo software (Version 10.8.2; BD Biosciences, Milpitas, CA, USA) to determine the percentage of CD11b^+^Iba-1^+^ cells and the mean fluorescence intensity (MFI).

#### 2.2.6. Measurement of Inflammatory Cytokines by Elisa

Cell culture supernatants were collected from all experimental groups. Levels of the inflammatory cytokines IL-1β, IL-6, and TNF-α were quantified using commercial ELISA kits in strict accordance with the manufacturers’ protocols. Cytokine concentrations were determined using kit-specific standard curves.

#### 2.2.7. Analysis of Oxidative Stress Markers

Cells or their corresponding culture supernatants were harvested. Levels of oxidative stress markers—including nitric oxide (NO), malondialdehyde (MDA), superoxide dismutase (SOD), and glutathione (GSH)—were measured according to the instructions of the respective commercial assay kits.

#### 2.2.8. Flow Cytometric Analysis of Ros and Apoptosis

ROS measurement: Cells were harvested, incubated with the DCFH-DA probe (as per kit instructions) at 37 °C for 20 min in the dark. Cells were then washed with PBS and promptly analyzed by flow cytometry (excitation/emission: 488/525 nm).

Apoptosis assay: Harvested cells were resuspended in Binding Buffer following the Annexin V-FITC/PI kit protocol. They were stained with Annexin V-FITC and PI for 15 min at room temperature in the dark, and flow cytometric analysis was performed within 1 h.

#### 2.2.9. Transmission Electron Microscopy for Mitochondrial Ultrastructure

Cells from each group were harvested and prefixed in 2.5% glutaraldehyde. After PBS rinses, they were post-fixed with 1% osmium tetroxide. Subsequently, samples were dehydrated through a graded acetone series, embedded in epoxy resin, and ultrathin-sectioned with an ultramicrotome. Sections were double-stained with uranyl acetate and lead citrate. Mitochondrial morphology was examined and imaged using a transmission electron microscope.

#### 2.2.10. Molecular Docking

Based on the component identification results, the three compounds with the highest Fragmentation Scores were selected as ligands. Molecular docking analyses were then performed with cGAS and STING as the receptor proteins. The 2D structures of these ligands were retrieved from the ChemSpider database. The receptor templates were high-resolution crystal structures of cGAS and STING, containing native ligands, which were retrieved from the PDB database. Water molecules were removed from these structures using PyMOL 2.5.2 for optimization. Using AutoDockTools 1.5.7, the receptor and ligand files were imported and preprocessed. This preprocessing included deleting water molecules, adding hydrogen atoms, and converting the structures to the pdbqt format. The docking parameters were set by defining a grid box. The center and dimensions of this box were specified to fully encompass the receptor’s binding pocket and the location of the native ligand, facilitating the identification of potential binding sites. All docking simulations were conducted using the software’s default parameters. The conformation exhibiting the lowest binding energy was recorded as the optimal docking pose. Finally, the optimal docking poses were visualized and analyzed with PyMOL 2.5.2. Ligand-receptor interaction diagrams were generated to elucidate the binding mechanisms.

#### 2.2.11. Western Blot Analysis

Total protein was extracted from cells of each group using RIPA lysis buffer. Protein concentrations were measured with a bicinchoninic acid (BCA) assay. Equal amounts of protein were separated by SDS-PAGE and subsequently transferred onto polyvinylidene difluoride (PVDF) membranes. Membranes were blocked with 5% skim milk for 1 h and then incubated overnight at 4 °C with gentle shaking in specific primary antibodies. The antibodies targeted Iba-1, iNOS, TNF-α, COX-2, cGAS, STING, and GAPDH, and were typically used at a 1:1000 dilution. Following three washes with Tris-buffered saline containing Tween 20 (TBST), membranes were incubated with horseradish peroxidase (HRP)-conjugated secondary antibodies (1:5000) for 1 h at room temperature. Protein bands were visualized using an enhanced chemiluminescence (ECL) substrate and detected with a chemiluminescence imaging system. GAPDH was used as the internal loading control. The band intensities of target proteins were quantified using ImageJ software (Version 1.54p; National Institutes of Health, Bethesda, MD, USA).

### 2.3. Statistical Analysis

All experiments were performed independently in at least three biological replicates, and data are presented as the mean ± standard deviation (SD). Statistical analyses were performed with GraphPad Prism (version 8.0). Normality and homogeneity of variances were assessed using the Shapiro–Wilk test and Bartlett’s test, respectively, and all datasets met the assumptions for parametric analysis (*p* > 0.05). Comparisons among multiple groups were performed using one-way analysis of variance (ANOVA), followed by Tukey’s post hoc test for pairwise comparisons. *p* < 0.05 was considered to be statistically significant.

## 3. Results

### 3.1. Chemical Composition of KSZZP

**Figure 1:**
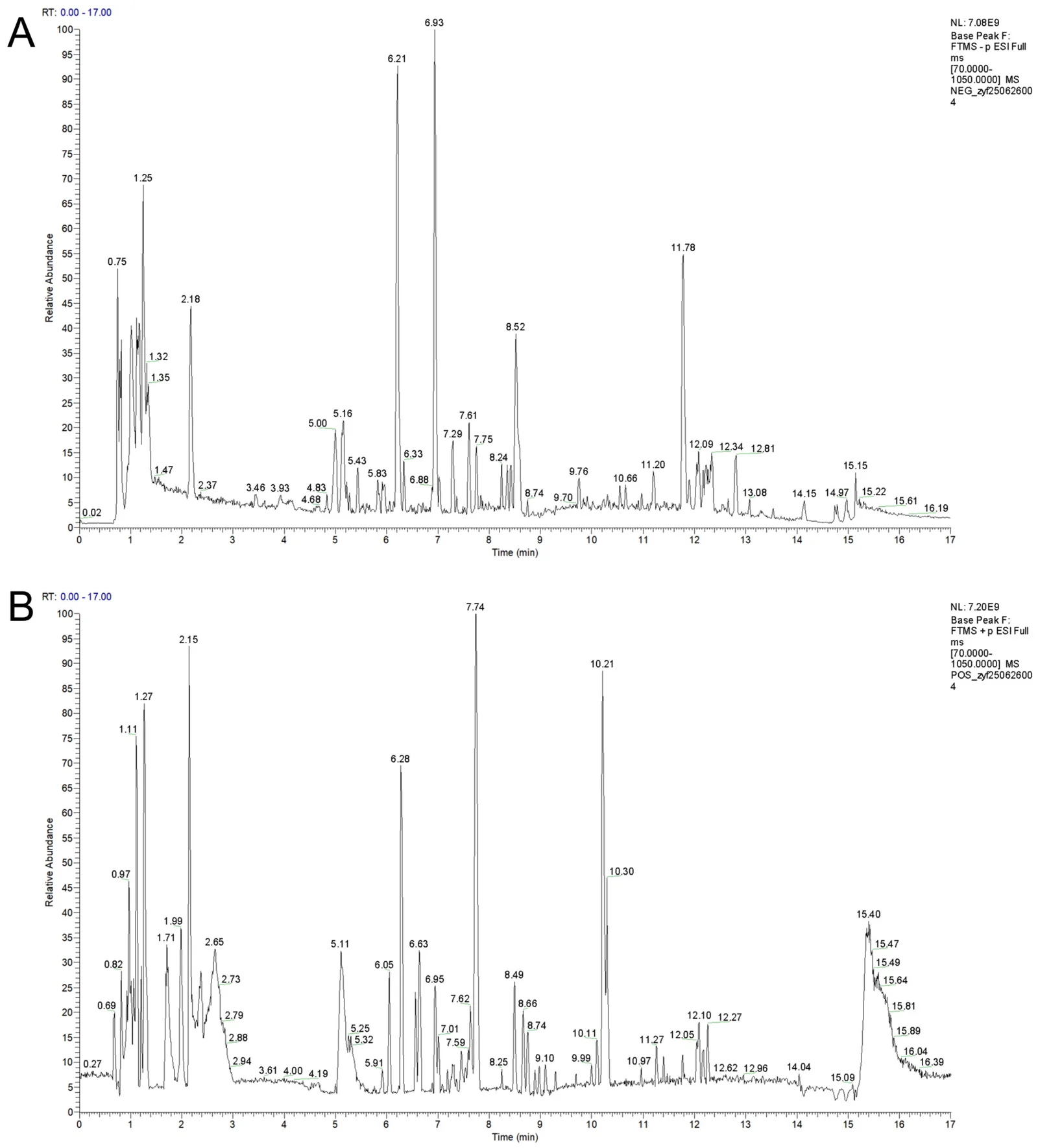
Total ion chromatogram (TIC) of KSZZP. (**A**) TIC in positive ion mode (ESI+). (**B**) TIC in negative ion mode (ESI-).

The chemical composition of KSZZP was systematically characterized using high-resolution mass spectrometry (HRMS) in both positive and negative ionization modes. A total of 67 compounds were identified in KSZZP (Table S1). These compounds belong to 26 distinct chemical classes, with the most abundant being flavonoids (12 compounds), prenol lipids (10), benzene and substituted derivatives (7), isoflavonoids (6), organooxygen compounds (5), cinnamic acids and derivatives (5), coumarins and derivatives (4), fatty acyls (3), linear 1,3-diarylpropanoids (3), phenols (2), and one compound each from the following classes: carboxylic acids and derivatives, imidazopyrimidines, purine nucleosides, tannins, benzopyrans, steroids and steroid derivatives, phenylpropanoic acids, hydroxy acids and derivatives, anthracenes, glycerophospholipids, and others. The total ion chromatogram (TIC) is presented in Figure 1A,B.

### 3.2. KSZZP Effects on Cell Viability and Safe Concentration Determination

**Figure 2:**
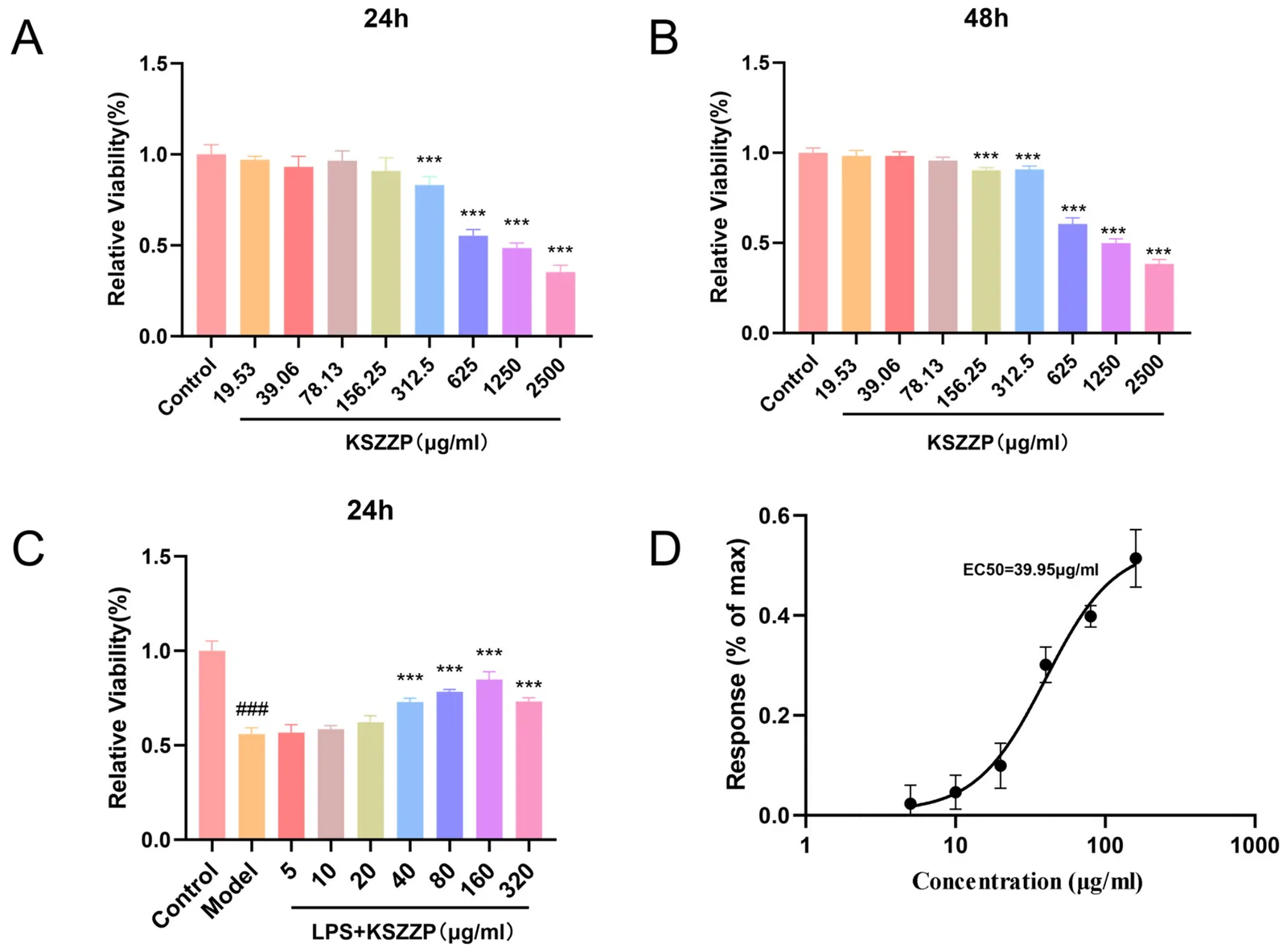
**Effects of KSZZP on BV-2 cell viability and the dose–response relationship**. (**A**) Effect of KSZZP on BV-2 cell viability (24 h). (**B**) Effect of KSZZP on BV-2 cell viability (48 h). (**C**) Cell viability after 24 h intervention with different concentrations of KSZZP in the LPS-induced inflammatory model. (**D**) Dose–response curve of KSZZP. Data are expressed as mean ± SD (n = 5). ^###^ *p* < 0.001 compared with the Control group; *** *p* < 0.001 compared with the Model group.

To determine the safe and effective working concentrations of KSZZP, we assessed its cytotoxicity using the CCK-8 assay. As shown in Figure 2A,B, at 24 h, concentrations ≥312.5 μg/mL significantly reduced BV-2 cell viability; whereas at 48 h, concentrations ≥156.25 μg/mL induced marked toxicity (*p* < 0.001). Accordingly, a 24 h treatment was selected for subsequent experiments, and concentrations were confined to the safe range. On the basis of these results, we further examined the protective effect of KSZZP in an LPS-induced inflammatory injury model. Gradient concentration experiments (Figure 2C) revealed that KSZZP exerted significant protective effects starting at 40 μg/mL. The protective effect increased in a concentration-dependent manner within the 40–160 μg/mL range and appeared to plateau at concentrations above 160 μg/mL. Nonlinear regression analysis yielded an EC_50_ of 39.95 μg/mL for KSZZP against LPS-induced injury (Figure 2D). This value was highly consistent with the threshold of 40 μg/mL, validating the use of this concentration as the minimum effective dose. Collectively, 40 μg/mL was chosen as the low dose (minimum effective concentration) and 160 μg/mL as the high dose (optimal effective concentration within the safe range) for all subsequent cellular functional experiments.

### 3.3. KSZZP Inhibits LPS-Induced Microglial Activation

**Figure 3:**
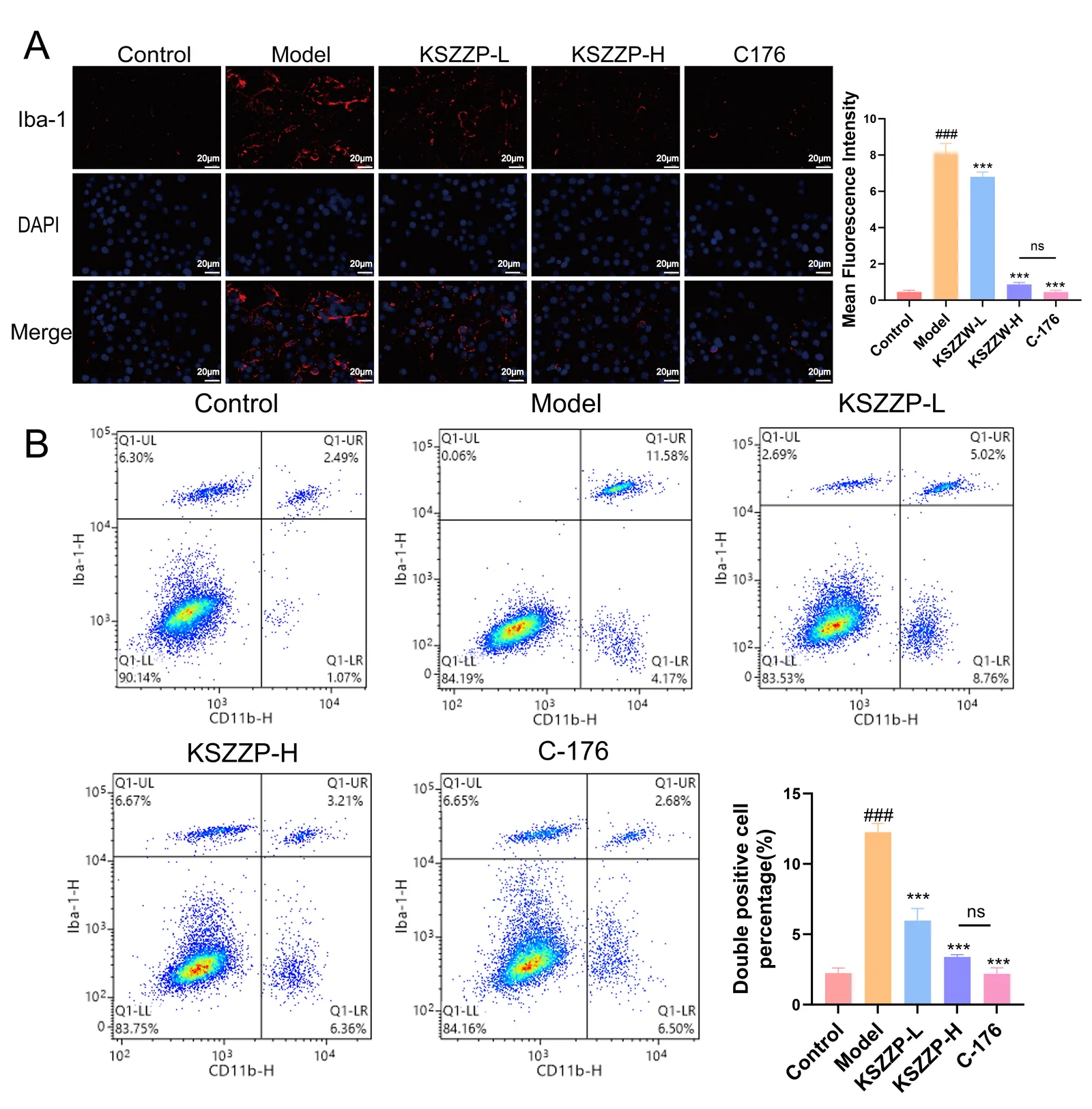
**Inhibitory effect of KSZZP on LPS-induced activation of BV-2 microglial cells**. (**A**) Representative immunofluorescence images of Iba-1 staining (scale bar: 20 μm; red: Iba-1, blue: DAPI; merge shows the overlay) and the quantitative analysis of the mean fluorescence intensity. (**B**) Representative flow cytometry scatter plots showing CD11b and Iba-1 double-positive cells and the quantitative statistics of their percentage. Data are expressed as mean ± SD (n = 3). ^###^ *p* < 0.001 compared with the Control group; *** *p* < 0.001 compared with the Model group; ns indicates no significant difference between the KSZZP-H and C-176 groups.

Microglial activation was first assessed by immunofluorescence staining for ionized calcium-binding adapter molecule 1 (Iba-1), a specific marker, followed by quantification of fluorescence intensity. Compared to the Control, the Model group showed significantly stronger Iba-1 fluorescence and a higher mean fluorescence intensity (Figure 3A; *p* < 0.001), confirming successful activation. Treatment with either KSZZP or the STING inhibitor C-176 significantly reduced Iba-1 fluorescence intensity compared to the Model group. To precisely quantify activated microglia, flow cytometry was performed on cells dual-labeled for CD11b and Iba-1. The percentage of CD11b^+^Iba-1^+^ cells was significantly higher in the Model group than in the Control group (Figure 3B; *p* < 0.001). KSZZP treatment dose-dependently reduced the proportion of CD11b^+^Iba-1^+^ cells, effectively suppressing microglial activation. Notably, the inhibitory effect of the KSZZP-H group was comparable to that of the C-176 group, with no significant difference between them. Together, these results demonstrate that KSZZP effectively inhibits LPS-induced microglial activation.

### 3.4. KSZZP Attenuates Inflammatory Cytokine Release and Oxidative Stress

**Figure 4:**
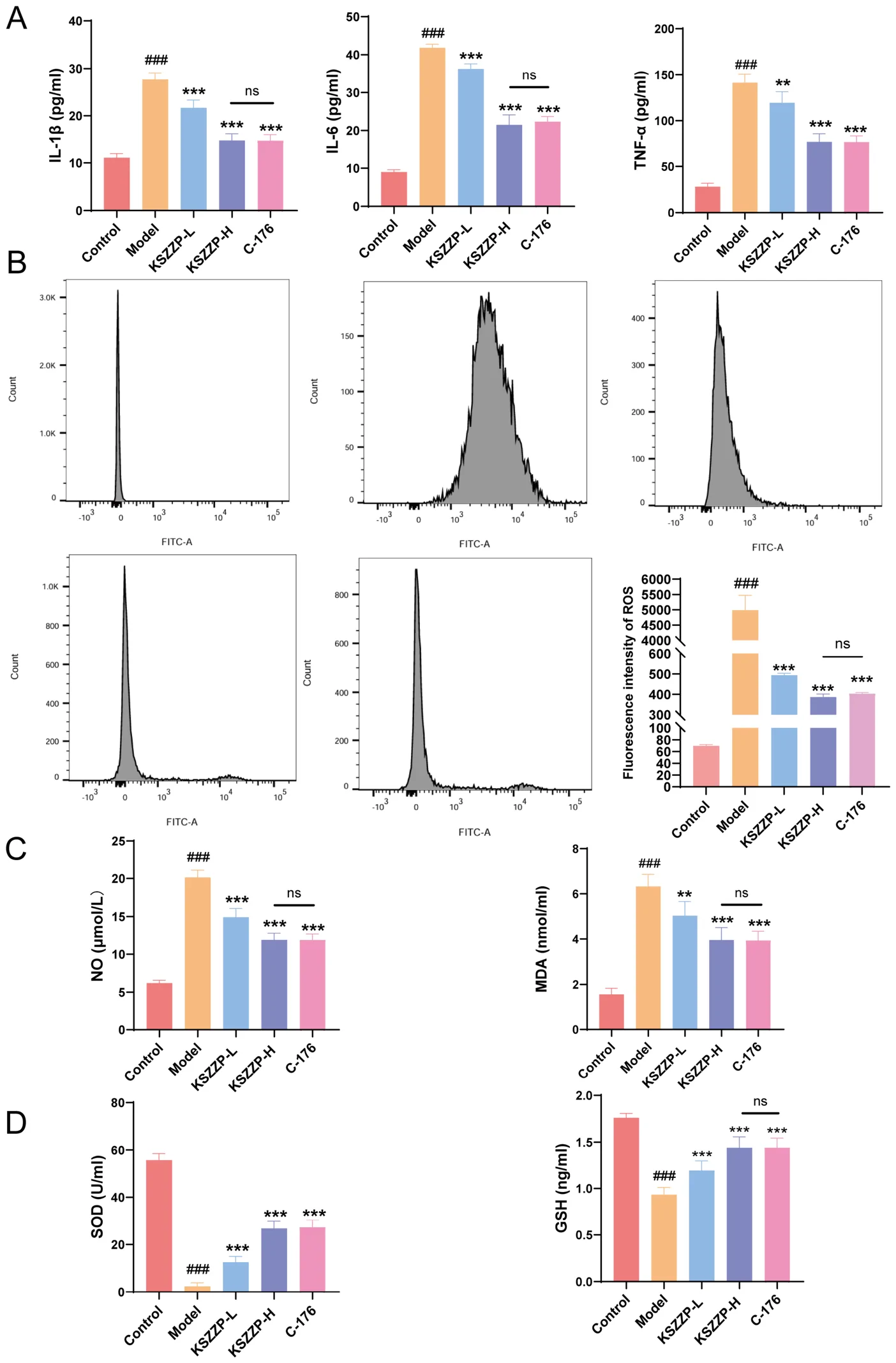
**Effects of KSZZP on inflammatory cytokines, ROS, oxidative damage, and antioxidant markers.** (**A**) Levels of IL-1β, IL-6, and TNF-α in different groups. (**B**) Representative flow cytometry plots of ROS (left) and quantitative analysis of fluorescence intensity (right). (**C**) Contents of NO and MDA. (**D**) Levels of SOD and GSH. Data are expressed as mean ± SD (n = 3). ^###^ *p* < 0.001 vs. Control group; ** *p* < 0.01, *** *p* < 0.001 vs. Model group; ns, not significant.

Compared to the Control, all measured inflammatory cytokines were significantly elevated in the LPS-treated (Model) group (Figure 4A; *p* < 0.001), confirming successful model induction. KSZZP treatment significantly and dose-dependently suppressed the release of these cytokines. Thus, KSZZP effectively inhibits LPS-induced cytokine release from microglia. As microglial activation and pro-inflammatory cytokine release are often associated with oxidative stress, we next assessed the impact of KSZZP on this process. Intracellular reactive oxygen species (ROS) levels and key oxidative/antioxidant markers were measured. Flow cytometry revealed a significant increase in intracellular ROS levels in LPS-treated cells compared to controls (Figure 4B; *p* < 0.001). KSZZP treatment dose-dependently reduced ROS levels. Assessment of oxidative stress markers showed that LPS treatment significantly increased nitric oxide (NO) and malondialdehyde (MDA) levels, while decreasing superoxide dismutase (SOD) activity and glutathione (GSH) content (Figure 4C,D; *p* < 0.001). KSZZP treatment reversed these changes, leading to decreased levels of ROS, NO, and MDA, and restored SOD activity and GSH content. Collectively, these findings demonstrate that KSZZP effectively inhibits LPS-induced ROS generation, alleviates oxidative damage, and enhances antioxidant capacity.

### 3.5. KSZZP Preserves Mitochondrial Ultrastructure in Microglia

**Figure 5:**
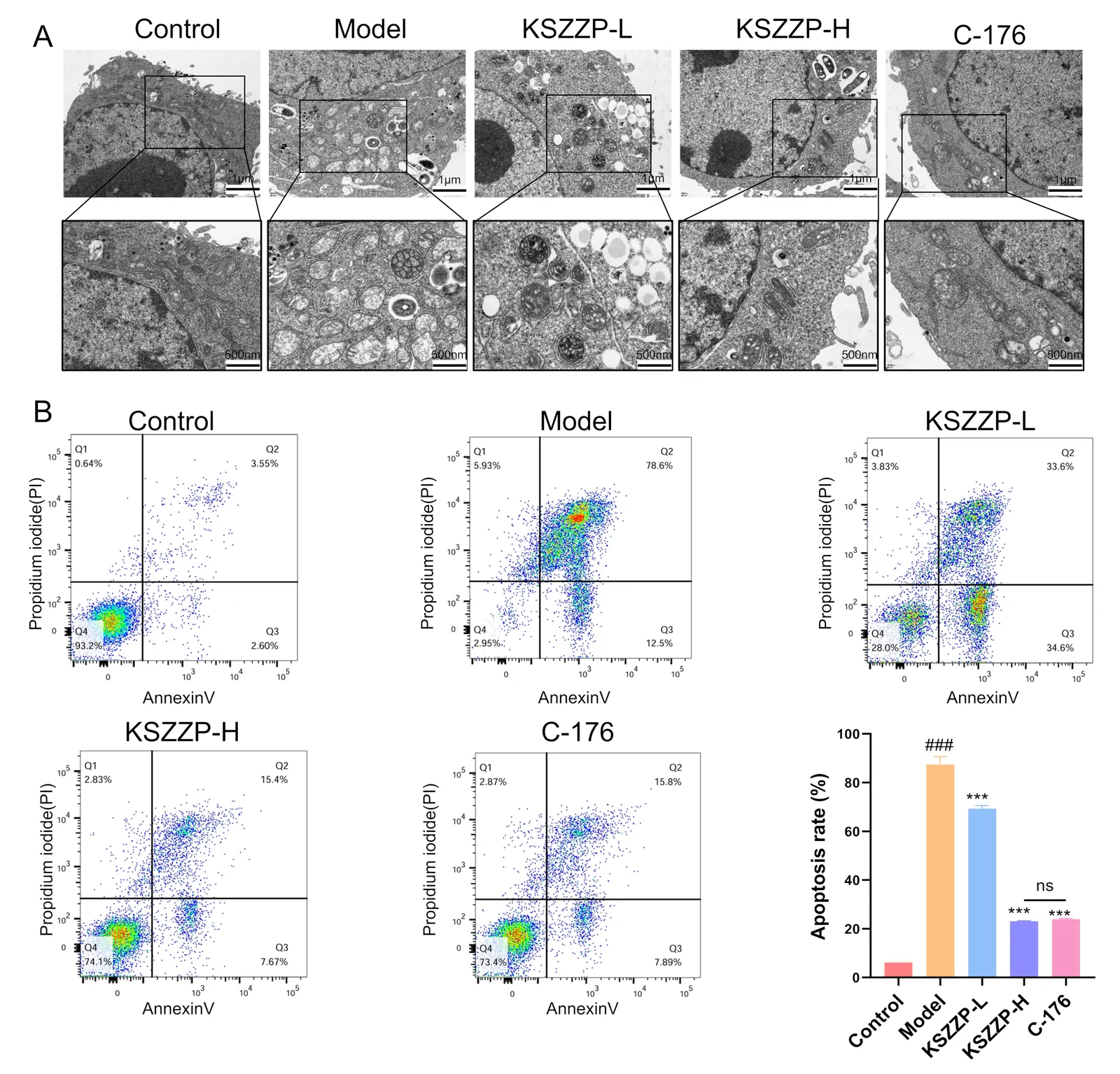
**KSZZP ameliorates mitochondrial ultrastructure and inhibits LPS-induced apoptosis.** (**A**) Representative transmission electron microscopy images of mitochondrial ultrastructure in each group. The upper panels show low-magnification fields (scale bar: 1 μm, 5000×), and the lower panels show corresponding high-magnification views (scale bar: 500 nm, 10,000×). (**B**) Flow cytometric analysis of apoptosis. The left panel shows Annexin V-FITC/PI scatter plots (Q1: necrotic cells; Q2: late apoptotic cells; Q3: viable cells; Q4: early apoptotic cells), and the right lower panel shows the quantitative statistics of the total apoptosis rate. Data are expressed as mean ± SD (n = 3). ^###^ *p* < 0.001 vs. Control group; *** *p* < 0.001 vs. Model group; ns, not significant between the KSZZP-H and C-176 groups.

To assess mitochondrial ultrastructure, transmission electron microscopy (TEM) was performed. Mitochondria in control cells displayed normal morphology, featuring intact membranes and orderly, densely packed cristae without evidence of swelling or vacuolization (Figure 5A). In contrast, LPS treatment induced significant mitochondrial damage, characterized by marked swelling, fragmentation or dissolution of cristae, and extensive vacuolization. KSZZP treatment markedly ameliorated these injuries, dose-dependently reducing swelling, partially restoring cristae structure, and decreasing vacuolization. The STING inhibitor C-176 elicited a similar protective effect, significantly alleviating swelling, restoring more regular cristae arrangement, and reducing vacuolization. These results indicate that KSZZP effectively mitigates LPS-induced damage to mitochondrial ultrastructure in microglia.

### 3.6. KSZZP Attenuates LPS-Induced Apoptosis

Apoptosis under inflammatory conditions was assessed using Annexin V-FITC/PI double staining. Flow cytometric analysis showed a significantly higher total apoptosis rate in the Model group compared to the Control group (Figure 5B; *p* < 0.001). Cell death was predominantly in the late apoptotic/necrotic stage, confirming successful induction of apoptosis by LPS. KSZZP treatment dose-dependently and significantly reduced the total apoptosis rate (*p* < 0.001). These results demonstrate that KSZZP effectively inhibits LPS-induced apoptosis in BV-2 cells. The anti-apoptotic effect of high-dose KSZZP was comparable to that of the STING inhibitor C-176.

### 3.7. Molecular Docking Identifies Potential cGAS-Sting Binders

**Figure 6:**
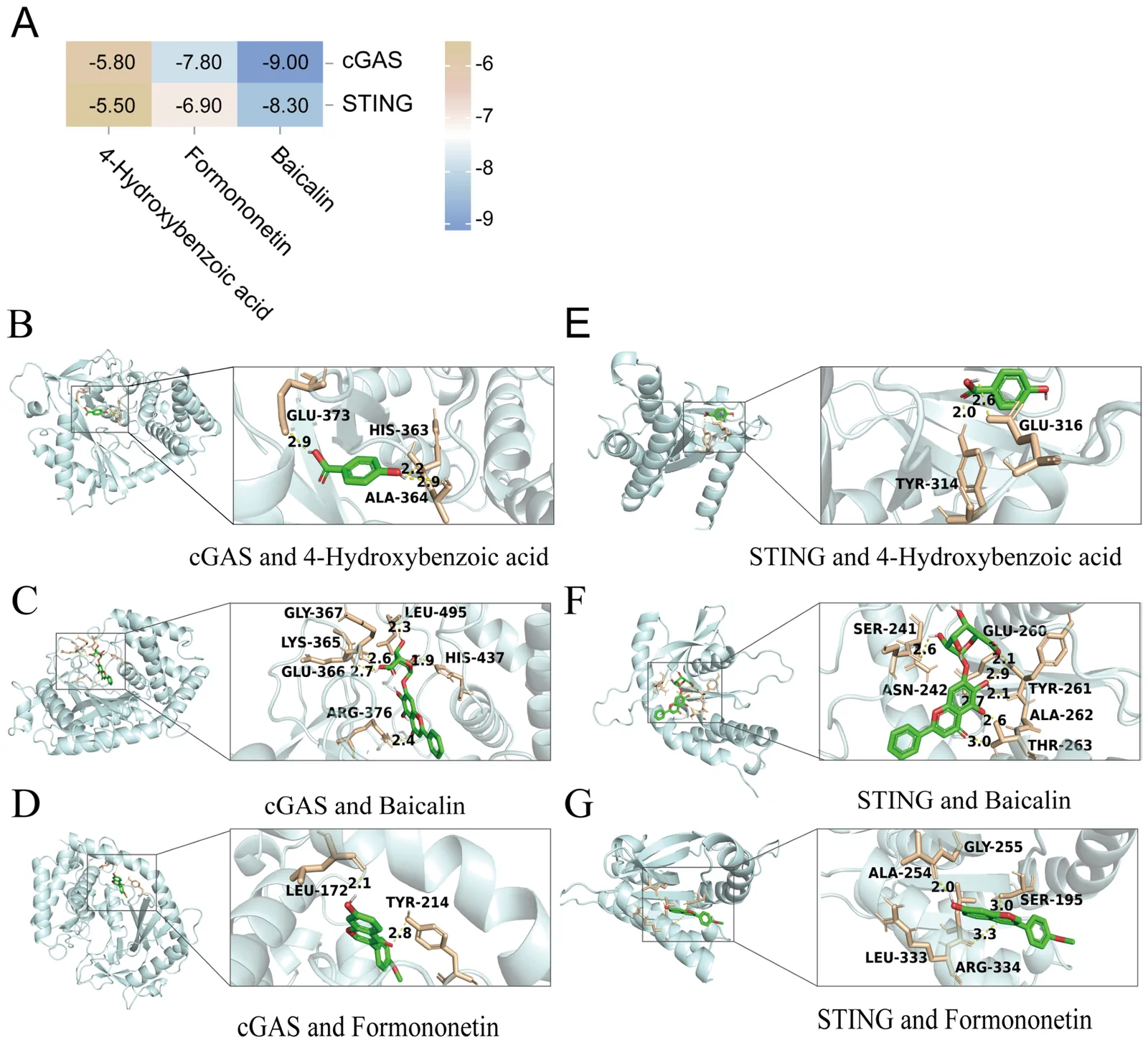
**Molecular docking analysis of candidate compounds with cGAS and STING proteins.** (**A**) Heatmap of the binding free energies (kcal/mol) of the three compounds to cGAS and STING. (**B**–**D**) Molecular docking poses of 4-Hydroxybenzoic acid, Formononetin, and Baicalin with cGAS protein. Insets highlight key hydrogen bond interactions. (**E**–**G**) Corresponding docking poses of the three compounds with STING protein, with insets showing key hydrogen bond interactions.

Molecular docking was performed to predict the interactions of the top three candidate compounds (by Fragmentation Score) with the cGAS and STING proteins. Lower binding free energy correlates with stronger ligand-receptor affinity and greater complex stability. All three compounds—4-Hydroxybenzoic acid, Formononetin, and Baicalin—showed favorable predicted binding affinity for both cGAS and STING, with binding free energies below −5.5 kcal/mol (Figure 6A). Each compound formed stable interactions within the protein active sites through networks of hydrogen bonds (Figure 6B–G). 4-Hydroxybenzoic acid formed hydrogen bonds primarily with cGAS residues HIS-363, ALA-364, and GLU-373, and with STING residues TYR-314 and GLU-316. Formononetin interacted with cGAS residues LEU-172 and TYR-214, and with STING residues SER-195, ALA-254, GLY-255, LEU-333, and ARG-334. Baicalin engaged a more extensive hydrogen bond network, involving six residues in cGAS (e.g., LYS-365) and six in STING (e.g., SER-241). These interactions suggest that these compounds have the potential to modulate the cGAS-STING pathway.

### 3.8. Kszzp Modulates the Cgas-Sting Pathway and Inflammatory Protein Expression

**Figure 7:**
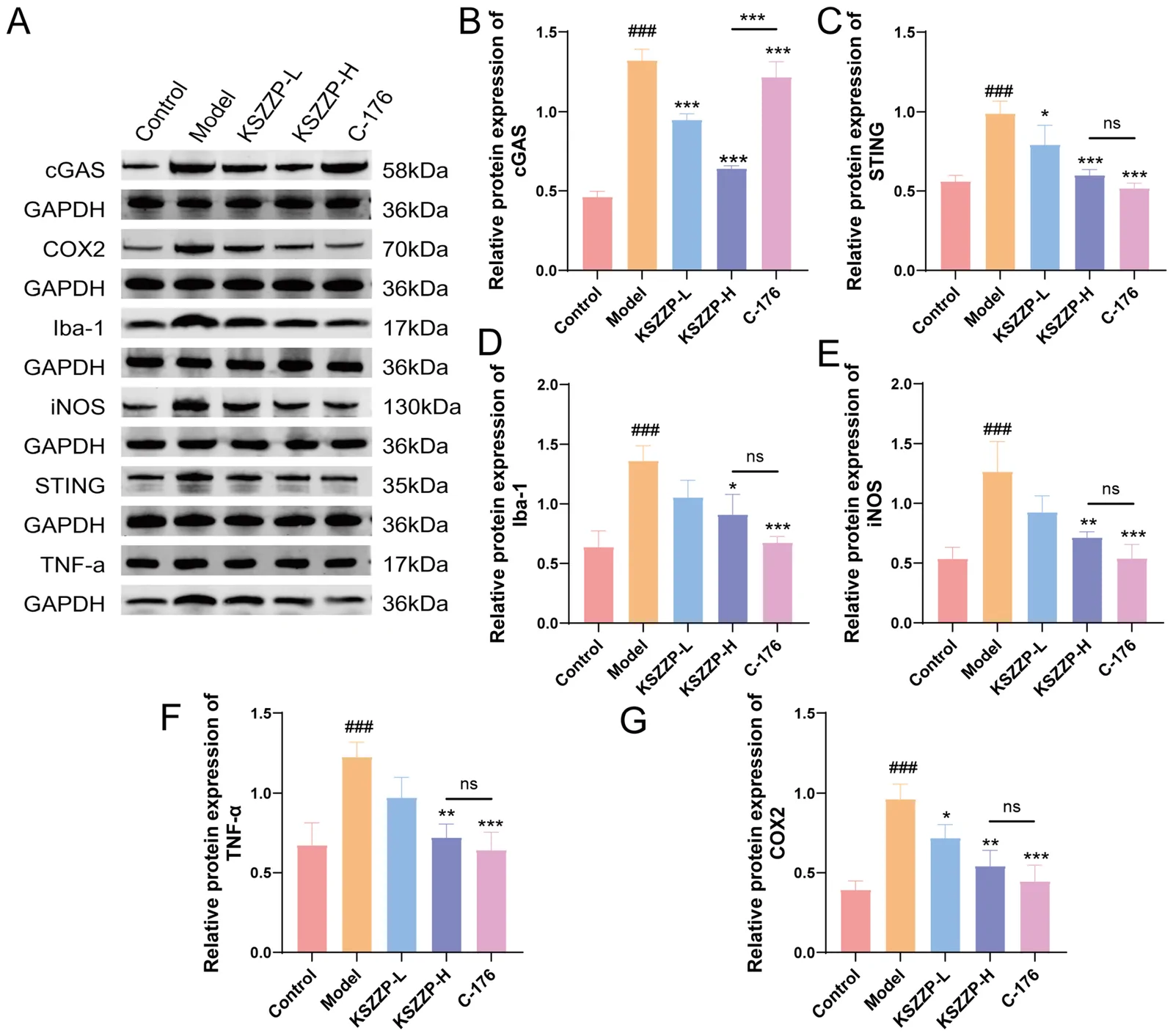
**KSZZP regulates the expression of cGAS-STING pathway components, microglial activation markers, and inflammatory proteins.** (**A**) Representative Western blot images showing protein expression across experimental groups. (**B**–**G**) Quantitative analysis of the relative expression levels for each protein. Data are expressed as mean ± SD (n = 3). ^###^ *p* < 0.001 vs. Control group; * *p* < 0.05, ** *p* < 0.01, *** *p* < 0.001 vs. Model group; ns, not significant between the KSZZP-H and C-176 groups.

To investigate the mechanism of KSZZP at the protein level, we analyzed the expression of cGAS-STING pathway components and related inflammatory proteins by Western blot. Compared to the Control group, protein levels of both cGAS and STING were significantly elevated in the Model group (Figure 7A). Levels of the microglial marker Iba-1, the M1 polarization marker iNOS, and the effector proteins TNF-α and COX-2 were also markedly increased (Figure 7B–G). KSZZP treatment dose-dependently downregulated the levels of cGAS, STING, iNOS, TNF-α, and COX-2. Notably, the STING-specific inhibitor C-176 did not significantly affect cGAS levels. In contrast, KSZZP downregulated cGAS and also effectively reduced the levels of downstream STING and inflammatory proteins (iNOS, TNF-α, COX-2). These findings suggest that the anti-neuroinflammatory effects of KSZZP may be associated with coordinated modulation of multiple key components of the cGAS-STING pathway.

## 4. Discussion

The accelerating aging of the global population has led to a significant rise in the incidence of neurodegenerative diseases, including Alzheimer’s disease (AD) and Parkinson’s disease (PD), posing a major public health challenge. Despite distinct clinical manifestations, these diseases share common pathological mechanisms. These include neuroinflammation, oxidative stress, mitochondrial dysfunction, and impaired DNA damage repair [27]. These pathological processes are interconnected in a complex network. Neuroinflammation serves as a central hub, where abnormal glial cell activation and cytokine release create a vicious cycle with oxidative stress and mitochondrial dysfunction, thereby collectively driving disease progression [28].

The cyclic GMP-AMP synthase (cGAS) is a cytosolic DNA sensor. Upon detecting DNA, it synthesizes the second messenger 2′3′-cGAMP, which activates the adapter protein stimulator of interferon genes (STING) and initiates an innate immune response [29]. In recent years, the cGAS-STING signaling pathway—a central mechanism linking cytosolic DNA sensing to innate immunity—has gained increasing attention for its role in neuroinflammation [30]. This pathway is activated by aberrant cytosolic DNA, such as that released from neuronal injury or mitochondrial stress. Its activation drives the expression of type I interferons and pro-inflammatory cytokines, playing a pivotal role in initiating and amplifying neuroinflammation, which makes it a promising therapeutic target for these diseases [31].

Given the complexity of neurodegenerative disease pathology, traditional Chinese medicine (TCM), with its characteristic multi-component and multi-target approach, offers a promising strategy to address this challenge. KSZZP is a modern preparation of a traditional formula used to “calm the mind and improve cognitive function.” It is commonly applied in clinical practice for cognitive dysfunction-related disorders. However, the molecular mechanisms underlying its anti-neuroinflammatory effects, particularly via microglial modulation, remain unclear. Therefore, in the present study, an LPS-induced BV-2 microglial inflammation model was used to examine the intervention effects of KSZZP on neuroinflammation and to investigate its underlying molecular mechanisms.

Defining the chemical composition is a prerequisite for elucidating the pharmacological mechanisms of TCMs. Accordingly, we first characterized the chemical profile of KSZZP using ultra-high-performance liquid chromatography coupled with Q-Exactive HFX mass spectrometry (UPLC-Q-Exactive HFX). A total of 67 compounds were identified. The predominant classes were flavonoids, prenol lipids, and isoflavonoids. Other detected classes included benzene and substituted derivatives, cinnamic acids and derivatives, and coumarins and derivatives. It should be noted that the chemical identification in this study was performed on the finished KSZZP product—a processed formulation of four herbs—rather than on individual crude herbs. Although UPLC-Q-Exactive HFX analysis enabled confident identification of 67 compounds based on high-resolution MS/MS data, the current experimental design does not allow definitive attribution of each compound to a specific herbal source in the formula. Such attribution would require parallel analysis of each constituent herb under identical analytical conditions. Baicalin, for instance, is not a characteristic constituent of any of the four individual herbs comprising the KSZZP formula, namely Tortoise Shell, Dragon Bone, *Acorus tatarinowii*, and *Polygala tenuifolia*. Its detection in KSZZP was based on a tentative structural assignment derived from MS/MS fragmentation information and does not conclusively confirm that the compound originates from the aforementioned herbal materials, nor can the possibility be excluded that it may arise from pharmaceutical excipients or from the transformation of other flavonoid precursors during the manufacturing process. These hypotheses warrant further validation using quantitative confirmatory methods, including authentic standard comparison and standard addition recovery experiments. Based on these findings, to further predict the binding affinity of potential active components in KSZZP to key proteins of the cGAS-STING pathway, we employed the fragmentation score as an objective screening criterion and selected the top three compounds—4-hydroxybenzoic acid, formononetin, and baicalin—from the 67 identified constituents as representative ligands for molecular docking against cGAS and STING. These three compounds not only exhibited the highest confidence in MS/MS spectral matching, thus ensuring the reliability of the structural information used for computational prediction, but also belonged to distinct chemical classes (benzoic acid derivatives, isoflavonoids, and flavonoid glycosides) with markedly different scaffolds, which facilitated a preliminary exploration of the binding modes of diverse compound types to the target proteins. Moreover, all three have been reported in the literature to possess anti-inflammatory or neuroprotective activities, which aligns closely with the focus of the present study [32,33,34]. Of note, the fragmentation score reflects structural confidence, not compound abundance (peak area). The molecular docking results demonstrated that all three compounds exhibited favorable predicted binding affinities to both cGAS and STING (binding free energies below −5.5 kcal/mol) and formed stable interactions within the protein active sites through hydrogen-bond networks, suggesting that these components may modulate the cGAS-STING pathway.

Inflammatory microglia are central to the pathogenesis of neurodegenerative diseases [35,36]. Notably, cGAS and STING are predominantly expressed in microglia. Their activation triggers robust pro-inflammatory responses in these cells, a response that is relatively weak in neurons and astrocytes [37,38]. This notion is further supported by studies in chronic neurodegenerative mouse models, which demonstrate that upregulation of cytosolic DNA sensors (e.g., cGAS) promotes microglial production of type I interferons and pro-inflammatory cytokines [39]. To model this process in vitro, we established a neuroinflammatory model by stimulating BV-2 microglial cells with LPS. LPS stimulation significantly increased the expression of the microglial activation marker Iba-1 and the release of the pro-inflammatory cytokines TNF-α, IL-6, and IL-1β, thereby recapitulating key pathological features of neuroinflammation observed in neurodegenerative diseases. KSZZP intervention induced a dose-dependent decrease in all measured indicators. This inhibitory effect paralleled that of the STING-specific inhibitor C-176, indicating that KSZZP effectively suppresses microglial overactivation and downstream inflammatory signaling.

LPS, a key component of the outer membrane of Gram-negative bacteria, is a classic inflammatory inducer. LPS triggers excessive inflammatory responses and exacerbates oxidative stress by dysregulating key molecules, including malondialdehyde (MDA), glutathione (GSH), and superoxide dismutase (SOD) [40]. Oxidative stress is closely linked to the onset and progression of neurodegenerative diseases [41]. Reactive oxygen species (ROS) play vital roles in physiological processes, including immune regulation and pathogen clearance. However, under pathological stress, excessive ROS accumulation leads to mitochondrial damage [42]. Our results align with these mechanisms. LPS stimulation of BV-2 microglia significantly increased levels of ROS, nitric oxide (NO), and MDA, while decreasing SOD activity and GSH content, indicating a state of oxidative imbalance. Transmission electron microscopy (TEM) revealed significant mitochondrial ultrastructural damage in LPS-treated cells, including swelling and cristae fragmentation. KSZZP intervention significantly ameliorated this oxidative/antioxidant imbalance and restored mitochondrial morphology and structure. Previous network pharmacology studies have predicted that KSZZP may exert neuroprotective effects via antioxidative stress pathways [43], and our experimental data on ROS, SOD, and GSH levels directly corroborate this prediction. Furthermore, KSZZP significantly inhibited LPS-induced apoptosis. Flow cytometry confirmed that LPS induced significant apoptosis, which was markedly attenuated by KSZZP treatment. Similarly, the modified KSZZP formula has been shown to inhibit apoptosis (as evidenced by upregulation of Bcl-2) in cerebral ischemia models, which is consistent with our Annexin V/PI results. This consistency suggests that anti-apoptotic activity may represent one of the pharmacological features shared by KSZZP-related formulas, although further studies are required to confirm this possibility [44]. In summary, KSZZP conferred protection against LPS-induced oxidative stress, mitochondrial damage, and apoptosis in microglial cells. It has been previously reported that the modified Kongsheng Zhenzhong Pills exerts anti-inflammatory effects by inhibiting the TLR4/NF-κB pathway, reducing hippocampal cytokine levels and promoting microglial M2 polarization [45]. This is complementary to our current finding that KSZZP downregulates M1 polarization markers, including iNOS, TNF-α, and COX-2.

Notably, Western blot analysis revealed that C-176, a direct STING inhibitor, did not affect upstream cGAS expression, whereas KSZZP dose-dependently downregulated the expression of cGAS, STING, and the downstream inflammatory proteins iNOS, TNF-α, and COX-2. This discrepancy suggests that KSZZP may act upstream of cGAS or directly target cGAS, rather than simply inhibiting STING function. Given that LPS-induced mitochondrial damage can trigger mtDNA release and activate cGAS, and considering the mitochondrial protection observed by TEM, KSZZP may reduce upstream activation signals for cGAS by mitigating mitochondrial damage. Alternatively, it may directly regulate cGAS expression through other transcriptional or post-transcriptional mechanisms. Future studies employing cGAS-specific inhibitors or gene knockdown experiments are warranted to further distinguish between these possibilities and to identify the precise targets.

The results of this study indicate that KSZZP effectively inhibits LPS-induced neuroinflammatory responses in microglial cells, with concomitant downregulation of cGAS and STING protein expression, suggesting that its anti-inflammatory effects may be associated with modulation of the cGAS-STING pathway. From a pharmacological perspective, these findings provide preliminary insights into the potential mechanism underlying the traditional use of KSZZP for “calming the mind and improving cognitive function” and offer an experimental basis for its further development in the prevention and treatment of neurodegenerative diseases.

Nevertheless, several limitations of this study should be acknowledged. First, our inference regarding the involvement of the cGAS-STING pathway is primarily based on correlative protein expression data and the literature linking mitochondrial stress to cGAS-STING activation; we neither directly examined cytosolic mtDNA accumulation nor assessed downstream phosphorylated activation markers (e.g., p-STING, p-TBK1, p-IRF3). Therefore, the current evidence for pathway activation remains incomplete, and our conclusions should be interpreted as preliminary indications rather than as confirmatory findings. Second, as this study was conducted in an in vitro cell model using the immortalized BV-2 microglial cell line, the main conclusions warrant further validation in animal models. Third, the KSZZP concentrations used in this study were based on the total weight of the commercial pill formulation (which contains honey as an excipient) rather than on purified herbal extracts. Thus, the reported concentrations represent the total extractable content of the formulation rather than the concentrations of individual active compounds. Honey itself contains various bioactive constituents, and its potential contribution to the observed effects cannot be ruled out. However, given the relatively low final concentration of the pill formulation in the culture medium, the honey excipient is diluted proportionally, and its impact on the experimental results is likely to be minimal. In addition, the molecular docking results only provide in silico predictions of structural binding, and the predicted interactions warrant further experimental validation using techniques such as surface plasmon resonance (SPR) or cellular thermal shift assays (CETSA). It should be emphasized that the fragmentation score reflects the confidence level in MS/MS-based structural identification rather than the biological activity, abundance, or pharmacological relevance of the compounds. Future studies should incorporate activity-guided fractionation or quantitative analysis to prioritize components with greater pharmacological relevance, and employ standardized herbal extracts or component-based fractionation strategies to unequivocally distinguish the contributions of herbal ingredients from those of excipients. Moreover, subsequent studies should combine in vivo animal models with pathway-specific interventions to further elucidate the regulatory effects of KSZZP on the cGAS-STING pathway and its causal relationship.

## 5. Conclusions

These findings indicate that KSZZP protects microglial cells against LPS-induced neurotoxicity, including excessive activation, neuroinflammation, oxidative stress, mitochondrial damage, and apoptosis, and that this protective effect may be associated with its inhibition of the cGAS-STING signaling pathway. From a pharmacological perspective, these results provide insights into the potential mechanism underlying the traditional use of KSZZP for “calming the mind and improving cognitive function,” suggesting that its anti-neuroinflammatory effects may involve multi-target modulation of the cGAS-STING pathway. Overall, this study provides preliminary experimental evidence to support the further development of KSZZP for the prevention and treatment of neurodegenerative diseases.

## Data Availability

The original contributions presented in this study are included in the article/Supplementary Materials. Further inquiries can be directed to the corresponding author.

## Supplementary Materials

The following supporting information can be downloaded at https://www.mdpi.com/article/10.3390/biomedicines14081665/s1. Table S1: Chemical Composition of KSZZP.
