# Supplementary material for: Effects of Kongsheng Zhenzhong Pills on Neuroinflammation and cGAS-STING Pathway Activity in BV-2 Microglia
Source: Biomedicines. 2026 Jul 24;14(8):1665. doi: 10.3390/biomedicines14081665 (PMC13509166; doi:10.3390/biomedicines14081665)
Supplement: Supplementary file 1 [file biomedicines-14-01665-s001.zip › biomedicines-4395186-supplementary.pdf]

Table S1 Chemical Composition of KSZZP

No.	Metabolite	Rt (min)	Mode	Adducts	*m/z*	Formula	Mass Error (ppm)	MS^2 fragmentation (*m/z*)	Fragmentation Score	Class	Relative abundance (Peak Area)
1	Cyclic N-Acetyl-D-mannosamine	0.88	neg	M+CH_3COO	280.1046	$C_8H_{15}NO_6$	3.74	118.0509,280.1020,190.0724,160.0617	89.5	Organooxygen compounds	161833.7187
2	6beta-Hydroxyipolamiide	0.95	pos	M+NH_4	440.1759	$C_{17}H_{26}O_{12}$	-0.72	422.1650,260.1127,242.1020,440.1759	88	Prenol lipids	6123769.458
3	Raffinose	0.95	neg	M+Cl, M+CH_3COO, M-H	503.1635	$C_{18}H_{32}O_{16}$	3.45	59.0138,71.0139,101.0243,179.0562	85.6	Organooxygen compounds	5648535.142
4	Yuheinoside	1.04	pos	M+NH_4-H_2O	360.1648	$C_{16}H_{24}O_9$	-2.95	360.1650,342.1542,181.1083,145.0494	86.4	Prenol lipids	3104163.407
5	Succinic acid	1.23	neg	M-H	117.0194	$C_4H_6O_4$	0.83	73.0295,117.0193,99.0086,116.9285	97.8	Carboxylic acids and derivatives	14418193.46
6	Adenine	1.31	pos	M+H	136.0617	$C_5H_5N_5$	-0.64	136.0616,119.0353,109.0510,94.0404	95.8	Imidazopyrimidines	21155311.39
7	ADENOSINE	2.58	pos	M+H	268.1036	$C_{10}H_{13}N_5O_4$	-1.65	136.0614,268.1031,202.5833,119.0348	98.6	Purine nucleosides	59520413.21
8	Protocatechuic acid	4.09	neg	M-H	153.0197	$C_7H_6O_4$	2.39	109.0294,153.0192,123.0452,108.0217	94.1	Benzene and substituted derivatives	8470675.025

9	Chlorogenic acid	4.99	neg	M-H	353.0895	$C_{16}H_{18}O_9$	4.81	191.0560,353.0836,352.1285,161.0240	85.7	Organooxygen compounds	718770.6626
10	Arillatose B	5.21	neg	M-H, M+Cl, M+CH3COO	517.1573	$C_{22}H_{30}O_{14}$	1.88	175.0401,160.0167,517.1555,193.0507	87.4	Cinnamic acids and derivatives	39451738.41
11	Sibiricose A6	5.28	neg	M-H, M+Cl, M+CH3COO	547.1671	$C_{23}H_{32}O_{15}$	0.42	205.0507,190.0272,547.1644,175.0040	96.3	Cinnamic acids and derivatives	31487804.86
12	Eupalitin 3-galactoside	5.32	neg	M+CH3COO	551.1417	$C_{23}H_{24}O_{12}$	2.24	431.0998,551.1396,461.1111,341.0671	87.3	Flavonoids	349273.0649
13	Caffeic acid	5.53	neg	M-H	179.0355	$C_9H_8O_4$	3.12	59.0139,135.0452,71.0139,179.0347	85.3	Cinnamic acids and derivatives	480213.7261
14	Sibiricose A5	5.54	neg	M-H	517.1583	$C_{22}H_{30}O_{14}$	3.94	175.0401,160.0168,517.1553,337.0938	85.8	Cinnamic acids and derivatives	2933240.231
15	Syringic acid	5.66	neg	M-H	197.0461	$C_9H_{10}O_5$	2.70	182.0221,197.0457,123.0087,166.9984	96.3	Benzene and substituted derivatives	3891798.47
16	Spinosin	5.98	pos	M+H	609.1812	$C_{28}H_{32}O_{15}$	-0.35	327.0856,297.0753,351.0856,429.1173	96.1	Flavonoids	5417423.855
17	Scopoletin	6.12	neg	M-H	191.0355	$C_{10}H_8O_4$	2.92	191.0345,148.0167,176.0118,59.0139	87.1	Coumarins and derivatives	34832.40486
18	10-Hydroxymajoroside	6.17	pos	M+H-2H2O	369.1174	$C_{17}H_{24}O_{11}$	-1.47	207.0650,175.0387,369.1172,147.0438	85	Organooxygen compounds	16253564.13
19	P-Coumaric acid	6.20	neg	M-H	163.0	$C_9H_8O_3$	2.30	119.0503,163.0381,	91.4	Coumarins and	816765.2715

				404			147.0453,148.0529		derivatives	
20	4-Hydroxybenzoic acid	6.21	neg	M-H	137.0245	$C_7H_6O_3$	0.78	93.0344,137.0243,108.0214,111.0087	99.4	Benzene and substituted derivatives	63777805.34
21	Lonfuranacid A	6.21	neg	M-H+H_2O	261.1348	$C_{12}H_{20}O_5$	4.39	261.1343,171.1028,243.1239,197.1183	85.7	Fatty Acyls	2012807.96
22	Ellagic acid	6.24	neg	M-H	300.9999	$C_{14}H_6O_8$	3.03	300.9989,299.9915,283.9960,245.0085	91.1	Tannins	349719.7944
23	Vanillic acid	6.33	neg	M-H	167.0354	$C_8H_8O_4$	2.73	108.0216,167.0352,152.0116,122.0611	93.2	Benzene and substituted derivatives	238612.3023
24	Vanillin	6.33	neg	M-H	151.0404	$C_8H_8O_3$	2.34	136.0166,151.0395,123.0089,59.0139	88.5	Phenols	2164156.169
25	Sibiricaxanthone B	6.46	neg	M-H	537.1268	$C_{24}H_{26}O_{14}$	3.35	267.0293,537.1247,256.0372,315.0505	89.2	Benzopyrans	1570413.253
26	Syringaldehyde	6.46	pos	M+H	183.0651	$C_9H_{10}O_4$	-0.67	123.0440,95.0494,155.0701,140.0466	89.4	Phenols	2821144.678
27	Naringenin chalcone	6.52	pos	M+H	273.0754	$C_{15}H_{12}O_5$	-1.14	153.0180,273.0741,147.0438,171.0285	85.1	Linear 1,3-diarylpropanoids	163547.1375
28	Cornuside	6.56	neg	M-H	541.1578	$C_{24}H_{30}O_{14}$	2.89	169.0143,541.1556,125.0244,379.1069	92.5	Prenol lipids	313273.7568
29	Baicalin	6.83	neg	M-H	445.0799	$C_{21}H_{18}O_{11}$	4.06	269.0455,241.0504,223.0402,175.0245	98.7	Flavonoids	4387388.394
30	Liguiritigenin-7-O-Beta-D-apiosyl-4'-O	6.85	neg	M-H, M+Cl	549.1630	$C_{26}H_{30}O_{13}$	3.06	255.0662,135.0087,119.0502,549.1617	92.1	Flavonoids	2891479.638

	-Beta-D-glucoside										
31	Isoliquiritin	7.04	pos	M+H	419.1332	$C_{21}H_{22}O_9$	-1.03	257.0802,137.0231,147.0438,239.0704	96.8	Flavonoids	6512359.207
32	Neoisoliquiritin	7.05	neg	M-H	417.1211	$C_{21}H_{22}O_9$	4.68	255.0663,148.0166,135.0088,417.1201	92.7	Flavonoids	5150945.407
33	4-Methoxysalicylic acid	7.11	neg	M-H	167.0355	$C_8H_8O_4$	2.89	108.0217,167.0352,123.0451,152.0112	95.3	Benzene and substituted derivatives	638586.8243
34	Ononin	7.14	pos	M+H	431.1334	$C_{22}H_{22}O_9$	-0.70	269.0801,254.0565,253.0489,237.0545	98.4	Isoflavonoids	3312304.98
35	Coagulin L	7.29	pos	M+H	651.3387	$C_{34}H_{50}O_{12}$	1.89	105.0335,136.0754,162.0910,651.3387	87.7	Steroids and steroid derivatives	3754587.165
36	Oroxylin A-7-O-Beta-D-glucuronide	7.48	pos	M+H	461.1074	$C_{22}H_{20}O_{11}$	-0.86	285.0751,270.0517,461.1096,269.0457	95.6	Flavonoids	2463311.212
37	Liquiritigenin	7.59	pos	M+H	257.0804	$C_{15}H_{12}O_4$	-1.85	137.0231,257.0805,147.0439,119.0492	95.6	Flavonoids	21727510.72
38	Populin	7.61	pos	M+Na	413.1221	$C_{20}H_{22}O_8$	3.50	147.0437,105.0336,119.0491,201.0551	87.4	Organooxygen compounds	126559053.1
39	Ferulic acid	7.74	pos	M+CH3OH+H	227.0911	$C_{10}H_{10}O_4$	-1.48	195.0650,227.0897,180.0420,152.0464	91.5	Cinnamic acids and derivatives	891450.9552
40	Calycosin	7.74	neg	M-H	283.0620	$C_{16}H_{12}O_5$	3.00	268.0376,283.0620,211.0405,239.0350	93.9	Isoflavonoids	210090.5363
41	Hydrocinnamic acid	7.95	neg	M-H	149.0612	$C_9H_{10}O_2$	2.39	149.0609,105.0710,59.0136	95.9	Phenylpropanoic acids	806951.4442

42	Retrochalcone	8.19	pos	M+H	271.0962	$C_{16}H_{14}O_4$	-0.96	121.0278,271.0965,229.0857,177.0541	88.2	Linear 1,3-diarylpropanoids	805189.9983
43	Eupafolin	8.34	pos	M+H	317.0653	$C_{16}H_{12}O_7$	-0.73	317.0647,302.0418,301.0334,285.0381	91.4	Flavonoids	516141.913
44	Malic acid	8.38	neg	M-H	133.0145	$C_4H_6O_5$	1.90	132.8678,115.0036,133.0139,71.0138	97	Hydroxy acids and derivatives	19444.53284
45	Traumatic Acid	8.52	neg	M-H	227.1295	$C_{12}H_{20}O_4$	2.51	183.1393,227.1289,165.1287,209.1183	88.2	Fatty Acyls	1412685.709
46	Tectorigenin	8.58	pos	M+H	301.0704	$C_{16}H_{12}O_6$	-0.92	301.0697,286.0464,285.0389,258.0514	95	Isoflavonoids	1971432.414
47	Iristectorigenin B	8.71	pos	M+H	331.0809	$C_{17}H_{14}O_7$	-0.97	331.0797,316.0571,301.0334,315.0489	87.7	Isoflavonoids	440071.8438
48	Isoliquiritigenin	8.74	neg	M-H	255.0666	$C_{15}H_{12}O_4$	1.26	119.0502,255.0665,135.0088,213.0546	96.2	Linear 1,3-diarylpropanoids	5013523.255
49	Curcumenol	8.79	pos	M+H	235.1691	$C_{15}H_{22}O_2$	-0.75	235.1699,217.1584,175.1484,133.1007	87.8	Prenol lipids	2909372.67
50	Formononetin	8.97	neg	M-H	267.0667	$C_{16}H_{12}O_4$	1.52	267.0662,252.0428,251.0354,223.0403	98.7	Isoflavonoids	663633.5569
51	Cotoin	8.98	neg	M-H	243.0669	$C_{14}H_{12}O_4$	2.40	243.0669,227.0350,242.1422,228.0418	86.1	Benzene and substituted derivatives	74728.05841
52	Hydramicromelin B	9.16	pos	M+H-H_2O	289.0702	$C_{15}H_{14}O_7$	-1.55	289.0701,228.0414,256.0363,274.0465	92.2	Coumarins and derivatives	7473894.05
53	Calealactone C	9.18	pos	M+CH_3CN	470.1	$C_{21}H_{26}O_8$	4.51	400.1746,442.1856,	87	Prenol lipids	976539.0935

				+Na	809			370.1281,385.1510			
54	Medicarpin	9.56	neg	M-H	269.0821	$C_{16}H_{14}O_4$	0.54	269.0819,254.0584,134.0372,89.0244	91.7	Isoflavonoids	213300.3364
55	Wogonin	9.63	pos	M+H	285.0755	$C_{16}H_{12}O_5$	-0.89	285.0752,270.0518,252.0419,269.0428	96.1	Flavonoids	1007036.177
56	Acacetin	9.64	neg	M-H	283.0620	$C_{16}H_{12}O_5$	2.91	268.0377,283.0623,163.0034,240.0423	93.2	Flavonoids	296108.2825
57	Nobiletin	9.76	pos	M+H	403.1386	$C_{21}H_{22}O_8$	-0.46	403.1374,373.0912,388.1145,330.0716	91.3	Flavonoids	365799.6746
58	Zedoarondiol	9.78	pos	M+H-H_2O	235.1691	$C_{15}H_{24}O_3$	-0.56	235.1698,217.1587,161.0986,133.1011	89.2	Prenol lipids	2514919.072
59	Arteannuin M	9.92	neg	M-H	267.1607	$C_{15}H_{24}O_4$	1.95	267.1587,223.1708,249.1494,205.1600	94.6	Prenol lipids	787885.6233
60	Alpha-Linolenic acid	10.31	pos	M+H_2O+H	297.2421	$C_{18}H_{30}O_2$	-3.27	279.2321,81.0703,95.0858,67.0547	90.8	Fatty Acyls	6223680.614
61	Emodin	10.74	neg	M-H	269.0460	$C_{15}H_{10}O_5$	1.63	269.0455,225.0560,241.0508,227.0347	95.5	Anthracenes	663373.7035
62	Gerberinside	10.92	pos	M+H-2H_2O	303.0859	$C_{16}H_{18}O_8$	-1.27	303.0854,287.0544,288.0612,259.0594	90.3	Coumarins and derivatives	2553518.307
63	Curcolonol	11.82	pos	M+H-H_2O, M+H	247.1327	$C_{15}H_{20}O_4$	-0.81	216.1142,247.1326,173.0958,201.0907	92.1	Prenol lipids	1076958.248
64	1-Stearoyl-sn-glycero-3-phosphocholine	12.82	pos	M+H	524.3713	$C_{26}H_{54}NO_7P$	0.39	184.0731,104.1071,524.3721,86.0968	92.4	Glycerophospholipids	857271.5056
65	alpha-Eudesmol	13.08	neg	M+HCOO	269.2127	$C_{15}H_{28}O$	2.09	269.2119,225.2227,251.2019,197.1914	88.7	Prenol lipids	956291.7759

66	Gossypol	13.56	neg	M-H	517.1891	$C_{30}H_{30}O_8$	4.37	517.1878,231.1027,471.1828,489.1932	93.3	Prenol lipids	1680073.565
67	Gallic acid	14.97	neg	M-H	169.0147	$C_7H_6O_5$	2.61	125.0244,169.0144,134.8655,107.0142	93.1	Benzene and substituted derivatives	2784942.625
